# Is Pollen Production of Birch Controlled by Genetics and Local Conditions?

**DOI:** 10.3390/ijerph19138160

**Published:** 2022-07-03

**Authors:** Surendra Ranpal, Miriam Sieverts, Verena Wörl, Georgia Kahlenberg, Stefanie Gilles, Maria Landgraf, Kira Köpke, Franziska Kolek, Daria Luschkova, Tobias Heckmann, Claudia Traidl-Hoffmann, Carmen Büttner, Athanasios Damialis, Susanne Jochner-Oette

**Affiliations:** 1 Physical Geography/Landscape Ecology and Sustainable Ecosystem Development, Catholic University of Eichstätt-Ingolstadt, 85072 Eichstätt, Germany; miriam-sieverts@web.de (M.S.); verena.woerl@gmx.de (V.W.); gkahlenberg@ku.de (G.K.); susanne.jochner@ku.de (S.J.-O.); 2 Environmental Medicine, Faculty of Medicine, University of Augsburg, 86156 Augsburg, Germany; stefanie.gilles@tum.de (S.G.); franziska.kolek@tum.de (F.K.); daria.luschkova@tum.de (D.L.); claudia.traidl-hoffmann@tum.de (C.T.-H.); dthanos@bio.auth.gr (A.D.); 3 Division Phytomedicine, Albrecht Daniel Thaer-Institute for Crop and Animal Sciences, Humboldt-Universität zu Berlin, 10099 Berlin, Germany; maria.landgraf@agrar.hu-berlin.de (M.L.); kira.koepke@agrar.hu-berlin.de (K.K.); carmen.buettner@agrar.hu-berlin.de (C.B.); 4 Department of Physical Geography, Catholic University of Eichstätt-Ingolstadt, 85072 Eichstätt, Germany; tobias.heckmann@ku.de; 5 Department of Ecology, School of Biology, Faculty of Sciences, Aristotle University of Thessaloniki, GR-54125 Thessaloniki, Greece

**Keywords:** *Betula pendula*, genotypes, reproduction, seed orchard

## Abstract

Intraspecific genetic variation might limit the relevance of environmental factors on plant traits. For example, the interaction between genetics and (a-)biotic factors regulating pollen production are still poorly understood. In this study, we investigated pollen production of 28 birch (*Betula pendula* Roth) individuals in the years 2019–2021. We sampled catkins of eleven groups of genetically identical trees, which were partially topped, but of the same age and located in a seed plantation in southern Germany characterized by similar microclimatic conditions. Furthermore, we monitored environmental factors such as air temperature, characterized air quality (NO_2_, NO_x_ and O_3_), and assessed potential solar radiation. We especially checked for differences between years as well as between and within clones and assessed the synchronicity of years with high/low pollen production. We present a robust mean for the pollen production of *Betula pendula* (1.66 million pollen grains per catkin). Our findings show temporal (H(2) = 46.29, *p* < 0.001) and clonal variations (H(4) = 21.44, *p* < 0.001) in pollen production. We conclude that synchronized high or low pollen production is not utterly site-specific and, in addition, not strictly dependent on genotypes. We suggest that appropriate clone selection based on application (seed plantation, urban planting) might be advantageous and encourage a long-term monitoring.

## 1. Introduction

Genetic variation among plant species is believed to limit the explanatory power of abiotic or biotic influential factors on certain plant traits. Several studies have revealed that plant traits, e.g., related to phenology, morphology, physiology, reproduction, and distribution are associated with genetic controls. Neophytou et al. [1] found a significant variation in the timing of bud burst among different Douglas fir progenies. Likewise, previous studies on poplar hybrids have reported that the patterns of tree biomass distribution above- and below-ground were genetically controlled [2,3]. Furthermore, naturally regenerated birch and aspen populations showed a variation between genotypes in the acclimatization to soil moisture conditions by altering biomass, root and leaf morphology, water potential, and gas exchange [4]. Rousi et al. [5] documented significant variations in intraspecific reproduction efficiency (anther residuals and seed production) among individuals of *B. pubescens* in two neighboring stands in Northern Finland. In addition, information on intraspecific genetic variations plays a crucial role to improve species distribution models [6]. Under varying environmental conditions, an exposed genotype has the ability to express phenotypic plasticity [7]. Studies on birch revealed phenotypic plasticity in leaf morphology of transplanted trees related to edaphic conditions [8] and larger phenotypic plasticity of juvenile above-ground growth traits in response to soil nutrient conditions [9]. Such findings indicate that traits of plant individuals of the same species growing under similar or different environmental conditions must be understood with the consideration of intraspecific variations.

Pollen are developed in anthers (angiosperms) or in microsporangia (gymnosperms) and their quantity per inflorescence is regarded as pollen production [10]. Pollen production may be controlled by the genes of taxa, species or varieties. It was suggested that the amount of pollen grains produced per anther and the number of anthers per flowers are genetically fixed and does not vary substantially [11,12,13]. In addition, any further variations could be related to changes in environmental conditions [12] such as meteorology, primarily air temperature [14,15,16], and edaphic factors [15,17], which alter the number of flowers and/or pollen production per flower. However, the role of these and other variables influencing pollen production are poorly known.

Most studies on pollen production of woody plants are limited to genera or species. Yet, a small number of studies have focused on the intraspecific level, for example, related to *Cupressus sempervirens* varieties [18] or *Theobroma cacao* clones [19]. Although Adams and Kunze [20] studied clonal variations in seed production in spruce, there has been little discussion on pollen production of genetically identical trees.

In general, genetically identical trees are preferentially used for various applications in science because it is assumed that they show the same behavior, e.g., related to phenology [21,22]. Long-term phenological observation networks such as the International Phenological Gardens in Europe (IPG) standardized phenological studies by establishing gardens with cloned plant individuals to exclude genetic effects [21,23]. Such phenological investigations based on cloned tree species assure that observed variances are due to environmental causes rather than genotypic differences between plants [24]. There have been attempts to explore the influences or exclusion of genetics on other pollen properties such as allergenicity. Ahlholm et al. [25] investigated the allergenicity of mountain birch pollen collected from trees of ten half-sib families growing in northern Finland and found that the concentration of the major birch pollen allergen (Bet v 1) is genetically controlled. In addition, concentrations of the allergen Cry j 1 produced by pollen of Japanese cedar were reported to be significantly different between trees of eight clones [26]. Similarly, Fernández-Caldas et al. [27] demonstrated considerable variations in pollen allergenicity (Ole e 1) of different varieties of *Olea europaea*.

However, studies related to pollen production compared for different clones in birch are lacking and are in general very sparse related to other species of the plant kingdom. Veilleux and Lauer [28] studied potato (*Solarium phurejas*) clones and suggested that plants of the same genotype respond similarly to the environment and produce the same amount of unreduced pollen grains. Panda et al. [29] observed a wide variation in pollen production per anther, pollen size and pollen viability among selected banana (*Musa* spp.) genotypes. Information on the variability of pollen production of genetically identical wind-pollinated plants is, however, largely lacking.

Detailed knowledge on the pollen production of a species is crucial for improving pollen forecasting [30]. Such forecasts have agronomical importance as seed production and, therefore, harvest outcomes often rely on pollen production [31]. Pollen production also plays a vital role in allergology. In the past few years, phenological, biometeorological, and aerobiological studies on allergenic plants have become more important due to the high prevalence of allergies around the world. According to the World Allergy Organization (WAO) up to 40% of the global population suffers from allergic sensitization [32], which could further increase by a parallel increase in pollen production [33,34,35,36].

Birch has a wide range of distribution in the Northern Hemisphere [37] and its pollen are highly allergenic [38,39] presenting a major source of allergic rhinitis in Europe [40]. Due to its aesthetic value, silver birch is a frequently used tree species in urban green space planning in Europe [41,42]. The abundance of birches, however, is problematic for many people who are allergic to pollen [43]. Studies on genotypic variations of pollen production of such allergenic tree species could identify clones, which are characterized by a lower pollen production. The breeding of such clones, e.g., for planting in urban green spaces, might also imply a reduction of atmospheric pollen concentration. On the other hand, seed plantations, in which a high pollen production of trees is desirable for a high quantity of seeds, may profit from those clones that are associated with a higher production of pollen. Most important, knowledge on the genetic variability of pollen production will allow for better evaluating the influence of environmental factors/climate change.

In this study, we assessed the pollen production of eleven groups of cloned weeping birch (*Betula pendula* Roth) individuals (*n* = 28) in three consecutive years (2019–2021). Since natural birch populations show a high grade of hybridization [44], we sampled inflorescences of genetically identical trees of the same age from a seed plantation (Baden-Württemberg, Germany), assessed the ambient microclimatic conditions and monitored any silvicultural treatments. We especially checked for differences between years as well as between and within clones and considered their synchronicity of pollen production levels. Based on the results, we discussed the implications of selecting clones producing a high/low level of pollen for seed plantations/urban planting.

## 2. Materials and Methods

**Figure 1:**
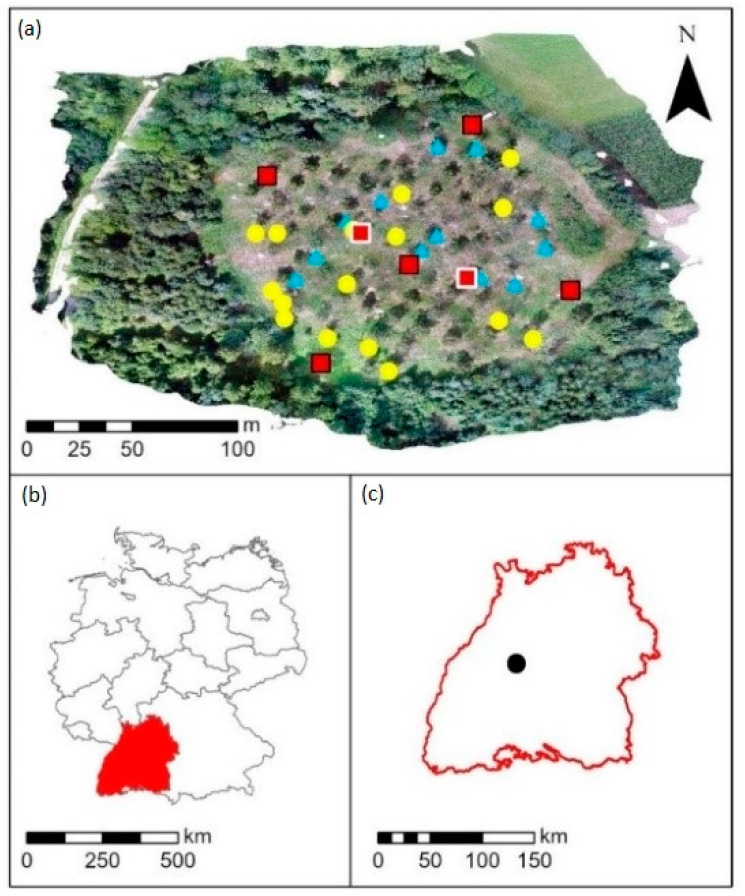
Study areas: (**a**) seed plantation near Wildberg (48°36′44″ N, 8°42′37″ E, 500 m a.s.l.) including measurement sites: blue triangles—topped sampled trees; yellow circles—non-topped sampled trees; red squares with black border—air temperature loggers; red squares with white border—passive samplers, (**b**) Baden-Württemberg in Germany (red fill) and (**c**) location in Baden-Württemberg (black circle).

For this study, we selected a birch seed plantation located near Wildberg (48°36′44″ N, 8°42′37″ E, 500 m a.s.l.) in Baden Württemberg, Germany (Figure 1). The average annual temperature is 8.6 °C and the precipitation sum is 892 mm (German Meteorological Service (DWD) station “Neubulach-Oberhaugstett”, 1991–2020 [45]). The plantation is located on a west-exposed slope with an inclination of approximately 2°−6° and the soil type is Cambisol [46]. This 1-hectare sized plantation was established in 2005 and additional birch trees were planted in 2012, resulting in a 7 m × 7 m seedling cluster, which is managed by Forst Baden-Württemberg (Forst BW; territory number 3, Nagoldtal). Initially, 215 trees belonging to 44 different clones were planted in a total of 13 rows and 17 columns. The clones and trees were randomized spatially throughout the site. Until now, almost half of the birch trees were removed as a thinning measure: 113 birch trees from 44 clones (with one to six individuals) are still present in the plantation.

The study was conducted in three successive years (2019–2021). We focussed on 28 trees, all planted in 2005, representing eleven clones from six different geographic origins (Table 1). These clone origins are, however, located nearby, within approximately 45 km to 130 km from the study site. The trees were selected based on the reachability of twigs and, therefore, inflorescences. The number of studied trees per clone, therefore, varied between one to four.

Male catkins were harvested in March after the beginning of catkin elongation and prior to anthesis. Samples were collected from different branches at 1.5 to 2 m above ground from all cardinal directions. In addition, we measured growth traits: (a) the perimeter at breast height, (b) the height of the tree and crown by use of Suunto PM-5/1520PC Height Meter, and (c) the crown diameter, which was calculated by averaging two perpendicular diameters of the crown at its widest portion.

We counted the number of catkins within a sampling cuboid (50 cm × 50 cm × 50 cm) in the crown, which was considered to characterize the average distribution of catkins in the tree [18]. We selected an ovoid shape of the crown to estimate pollen production per tree.

In July 2018, tree topping (cutting of the apical parts of the main trunk), which is an intended measure to increase seed production [47], was carried out in the seed plantation. Therefore, the sampled trees were categorized as topped (*n* = 12) and non-topped (*n* = 16). Six more sampled trees were topped in July 2020; however, male catkins were already formed in those trees and, therefore, no large effect on pollen production was assumed.

Air temperature and precipitation data were obtained from a 5 km distant DWD climate station “Neubulach-Oberhaugstett” [45]. In addition, we installed five temperature loggers (HOBO Pro v2 U23-001, Onset, Bourne, MA, USA) from spring 2019 (8 April) until summer 2021 (20 June). One logger was installed in the center and four at the northern, eastern, southern, and western borders of the plantation (red squares with black border in Figure 1) to determine temperature differences within the site. Each logger was placed in a radiation shield and mounted at a height of 2 m at the northern side of a birch tree. The loggers’ data were retrieved and processed using HOBOware (Version 3.7.23) from Onset, Bourne, MA, USA.

The air quality of the study site was characterized by the measured values of nitrogen dioxide (NO_2_), nitrogen oxides (NO_x_) and ozone (O_3_) concentrations monitored directly at the stem of the birch trees (*n* = 2, red squares with white border in Figure 1). Passive sampling of these pollutants lasted one week in summer 2020 (25 June to 2 July). The passive samplers were supplied and evaluated by Passam AG (Männedorf, Switzerland).

For estimating the potential solar radiation around each tree, the unmanned aerial vehicle Phantom 4 Pro, DJI, Nanshan, Shenzhen, China was used, which features an onboard RGB camera with a sensor resolution of 12 megapixels and a focal length of 24 mm. The flight altitude was 35 m above ground level. During the flight, which took place on 10 August 2019 and lasted approximately 17 min, 712 photos were taken with an overlap of 80%. A digital elevation model was generated using Metashape Professional (Version 1.8.1) from Agisoft LLC, St. Petersburg, Russia. In ArcGIS Pro (version 2.7.0) software from ESRI, Redlands, CA, USA, the spatial analyst tool “Solar radiation (area)” was used to calculate the potential solar radiation (W/m^2^) on the surface depending on the time of day and position of the sun as well as the latitude for each pixel of the digital elevation model. We calculated solar radiation for each pixel as a sum for the period 1 May until 31 August as this period is critical for the start and development of the following year’s catkin [48]. We selected a buffer of two meters around each tree and calculated the mean solar radiation. We assume only minor differences in the canopy of the surrounding forest and, therefore, use the data gained in 2019 for a general site characteristic for the whole study period.

To extract pollen grains, we adapted the method proposed by Damialis et al. [18]. For each year, one average-sized inflorescence from each cardinal direction and per tree was selected, its length and width were measured (at the widest point), and the number of flowers was counted. Then, each catkin was soaked in a 10% KOH solution [31,49] and boiled at 120 °C the following day. Afterwards, the plant material was crushed with a glass rod to break up plant tissues and to allow pollen release. To prevent pollen clumping [50], we added glycerol (70%), a bipolar solvent, to a volume of 20 mL; safranin was added as a stain. Two aliquot samples (10 µL each) per suspension were obtained using a VITLAB^®^ micropipette while stirring it vigorously to ensure homogeneity. Subsequently, the extraction was put on microscope slides and covered with slips. Pollen grains on these slides were subsequently counted at 100× magnification (Zeiss AXIO Lab.A1, Germany). In case of a large difference between the pollen counts obtained from these two slides (>30%), the procedure was repeated in order to increase the homogeneity of the suspension.

We estimated pollen production at various scales [18]: The number of pollen grains per catkin (*P_ca_*) was calculated using Equation (1):

(1) $$P_{ca}=\frac{V_{su}}{V_{sa}}p$$

where $V_{su}$ and $V_{sa}$ are the volumes of the suspension (in mL) and the sample taken (in µL), respectively, and *p* is the number of pollen grains counted per 10 µL solution.

The number of pollen grains per flower ($P_{fl}$) was estimated as follows (Equation (2)):

(2) $$P_{fl}=\frac{P_{ca}}{fl}$$

where *fl* is the number of flowers per catkin.

The number of pollen grains per volume unit (m^3^) of crown ($P_{cr}$) was estimated using Equation (3):

(3) $$P_{cr}=P_{ca}\;\frac{C_{su}}{M}$$

where $C_{su}$ is the number of catkins per crown sampling unit (cuboid) and M is the volume of the sampling unit.

The number of pollen grains per individual ($P_{in}$) was estimated using Equation (4):

(4) $$P_{in}=P_{cr}V$$

where $P_{cr}$ is the number of pollen grains per crown volume unit (see Equation (3)) and V is the total volume (in m^3^) of the crown. The volume of an ovoid tree Crown can be calculated as follows (Equation (5)):

(5) $$V=\frac{\pi d_{1}d_{2}h_{c}}{6}$$

where π ≈ 3.14, $d_{1}$ and $d_{2}$ are two perpendicular diameters of the crown, at its widest part, and $h_{c}$ is the crown height.

Pollen production per flower, catkin, and volume unit of crown, as well as flowers per catkin and catkins per crown sampling unit, were descriptively analyzed. These reproductive metrics were non-normally distributed according to Shapiro–Wilk test. We checked for differences among sampling years and clones using the Kruskal–Wallis test and post-hoc (Dunn) test. Correlation analyses between the reproduction metrics and between solar radiation and pollen production were conducted using Spearman’s correlation test. The differences between topped and non-topped trees were analyzed using Mann–Whitney U test. The variation within non-topped clones was assessed by comparing the coefficient of variances (CVs). For indicating if one specific clone can be proposed as “good” or “poor” regarding pollen production, we averaged the crown metrics (crown height and crown width) of all non-topped trees and calculated a mean crown volume. We considered that this computed crown dimension would represent an average non-topped birch tree in the seed plantation. Similarly, we calculated the mean *P_ca_* and mean *C_su_* obtained from the non-topped trees during the study years. These values allowed us to quantify the total *P_in_* for an average tree (using Equation (5)). Further, we used average *P_ca_* and *C_su_* of each clone along with the crown volume of an average tree to calculate the pollen produced by each clone under mean growth parameters to compare the pollen produced by each clone to an average birch tree. All statistical analyses and visualizations were performed in RStudio (version 4.1.2) from RStudio, PBC, Boston, MA, USA, ArcGIS Pro (version 2.7.0) or Microsoft Excel 2016 from Microsoft, Washington, DC, USA.

## 3. Results

### 3.1. Descriptive Statistics and Correlation Analyses among Reproductive Metrics

The average pollen production per catkin (*P_ca_*) for all selected 28 trees and all study years (2019–2021) was 1.66 ± 1.28 million pollen grains (see Table 2). *P_ca_* varied within a wide range from 48,000 to 8.27 million pollen grains, especially in the year 2019. *P_ca_* in 2020 was 11% higher and 28% lower compared to 2019 and 2021 when regarding mean values. *P_ca_* in 2021 was 54% higher compared to 2019.

The number of catkins in a crown sampling unit (*C_su_*; 0.125 m^3^) ranged between 3 and 120 with an average of 29 catkins. *C_su_* in 2020 were 191% and 232% higher compared to 2019 and 2021 and 17% lower in 2021 compared to 2019. Statistics for all analyzed levels (*P_ca_*, *P_fl_*, *P_cr_*, *fl* and *C_su_*) are presented in the Appendix A (Table A1).

**Figure 2:**
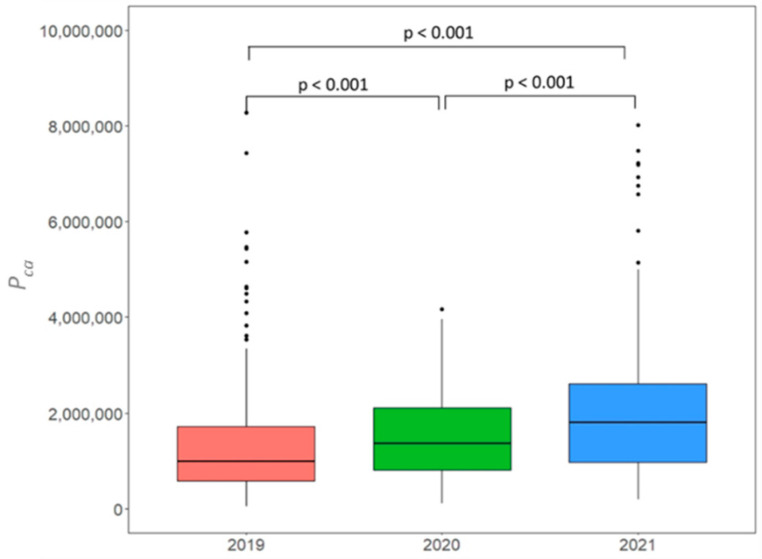
Boxplots based on pollen production per catkin (*P_ca_*) (eight replications per tree) estimated for 28 trees in the seed plantation in Wildberg for 2019, 2020 and 2021. The interquartile range is represented by the height of the boxes, maximum and minimum values by the upper and lower whiskers, the median by bold horizontal lines in the boxes, points indicate outliers, lines above boxplots indicate pair of years, which were significantly different (Kruskal–Wallis test and Dunn’s multiple comparison’s tests).

We detected a statistically significant difference between *P_ca_* among the three study years (H (2) = 46.29, *p* < 0.001). A post-hoc test revealed that there were significant differences between all pairs of years (Figure 2). The same applied for *C_su_* (H (2) = 200.78, *p* < 0.001; boxplots not shown).

Correlations between different reproductive metrics from all study years are shown in Table 3. The highest Spearman’s correlation coefficient was found for *P_fl_* and *P_ca_* (*r_s_* = 0.980, *p* < 0.001). *fl* was associated with a negative correlation with *P_fl_* (*r_s_* = −0.230, *p* < 0.001) and a positive correlation with *C_su_* (*r_s_* = 0.200, *p* < 0.001). *P_fl_* or *P_ca_* did not show any significant correlations with *C_su_*.

The temporal variations of pollen production (Table 2, Figure 2) probably include some abiotic and biotic influential factors, which are described below.

### 3.2. Meteorological Differences in the Study Years

**Figure 3:**
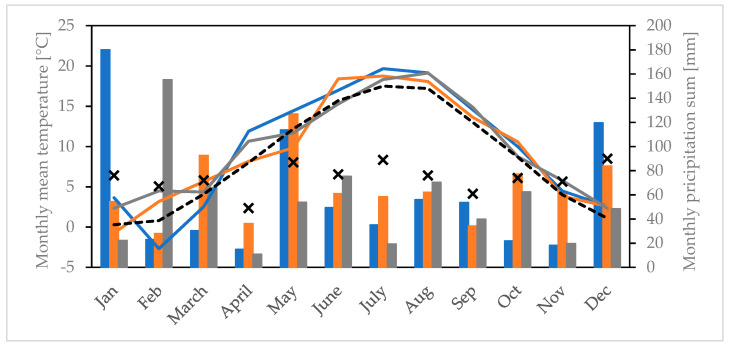
Monthly average temperature (lines) and monthly precipitation sum (bars) for the years 2018 (blue), 2019 (orange) and 2020 (grey) recorded at a nearby weather station (DWD station Neubulach-Oberhaugstett). *x*-axis: months, left *y*-axis: monthly mean temperature in °C, right *y*-axis: monthly precipitation sum in mm. Mean values (1991–2020) are displayed as black dashed lines (temperature) and crosses (precipitation).

Figure 3 shows the meteorological conditions at Neubulach-Oberhaugstett, near the plantation site, for the period 2018–2020. In addition, we calculated averages for months that are especially important for the initiation and formation of catkin of the following year (i.e., May until August of the preceding summer; [48]). We estimated the lowest average *P_ca_* in 2019 (Table 2, Figure 2), which was following a relatively high temperature (17.6 °C) and moderate precipitation (63.5 mm) during those specified four months in 2018 (compared to 2019 and 2020). Mean *P_ca_* was higher in 2020 and linked to a preceding period with a moderate temperature (16.3 °C), but a high precipitation sum (77.1 mm) was recorded during May–August 2019. The average numbers of pollen grains per catkin estimated in 2021 was the highest among all study years; the preceding period in 2020 was associated with the lowest temperature mean (16.1 °C) and precipitation sum (54.7 mm) compared to 2018 and 2019. The selected period of the year was on average warmer but received less precipitation in all study years compared to 1991–2020 (15.7 °C; 82.3 mm).

Site-specific temperature data (8 April 2019 to 20 June 2021) at five different locations within the plantation (see Figure 3) were found to be not significantly different according to ANOVA tests (daily mean temperature: F (4, 4020) = 0.73, *p* = 0.570, monthly mean temperature: F (4, 125) = 0.03, *p* = 0.990). In addition, air pollutants sampled at two sites (see Figure 1) were almost identical: site 1—NO_2_ < 6.5 µg/m^3^, NO_x_ = 2.4 µg/m^3^ and O_3_ = 33.2 µg/m^3^; site 2—NO_2_ < 6.5 µg/m^3^, NO_x_ = 2.6 µg/m^3^ and O_3_ = 36.2 µg/m^3^.

**Figure 4:**
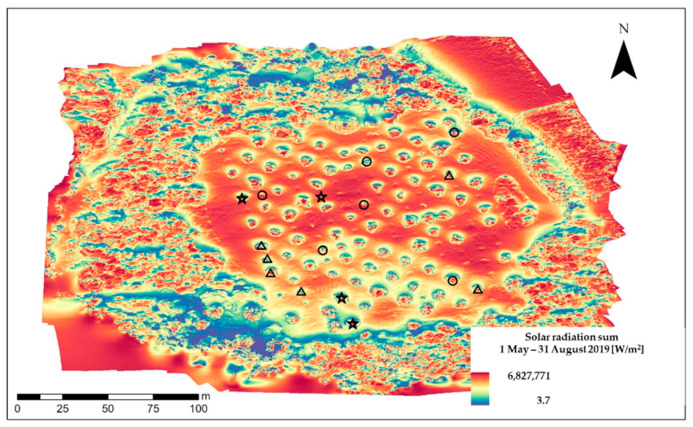
Solar radiation map and synchrony of pollen production levels of non-topped trees (*n* = 16). Circles—group 1 (*n* = 6, trees with maximum *P_ca_* in 2020); triangles—Group 2 (*n* = 4, trees with *P_ca_* extraordinarily high in 2021) and stars—group 3 (*n* = 6, almost constant *P_ca_* values and/or minimum values in 2020).

Incoming shortwave radiation, expressed as the sum of radiation in the months May to August in W/m^2^, varies within the seed plantation due to the surrounding forested area and is generally lower in the southern part (Figure 4). However, we found no statistically significant correlation between mean *P_ca_* (2019–2021) and solar radiation (*r_s_* = −0.111, *p* = 0.574) when regarding all 28 selected birch trees. For single years, we detected an alternating (but still not significant) signal: in 2019 and 2020, the correlations were positive (*r_s_* = 0.201, *p* = 0.304 and *r_s_* = 0.076, *p* = 0.702, respectively) and in 2021 the correlation was negative (*r_s_* =−0.149, *p* = 0.4489).

### 3.3. Tree Condition

The differences in pollen production between trees that were topped in 2018 and non-topped trees were compared for 2020 and 2021 (Table 4). Pollen production in 2019 was considered unaffected by tree topping since this intervention was carried out after the formation of catkins.

Mann–Whitney U tests revealed that there were significant differences between topped compared to non-topped trees. The first year with potential effects of tree topping (2020) was associated with a significantly lower pollen production and a significantly higher flower and catkin formation compared to non-topped trees. For example, *P_ca_* was 27% lower, *P_fl_* was 34% lower, *fl* were 9% higher and *C_su_* were 40% higher for these damaged trees. The effect of tree topping was most obvious in 2021 since all metrics were associated with significantly higher mean values. For example, *P_ca_* was 5% higher, *P_cr_* was 70% higher, *fl* were 5% higher and *C_su_* were 44% higher for topped compared to non-topped trees. In 2021, the effect on *P_cr_* was most pronounced, especially when bearing in mind that this last study year presented a year with poor catkin formation (see Table 2).

### 3.4. Synchrony of Pollen Production Levels

**Figure 5:**
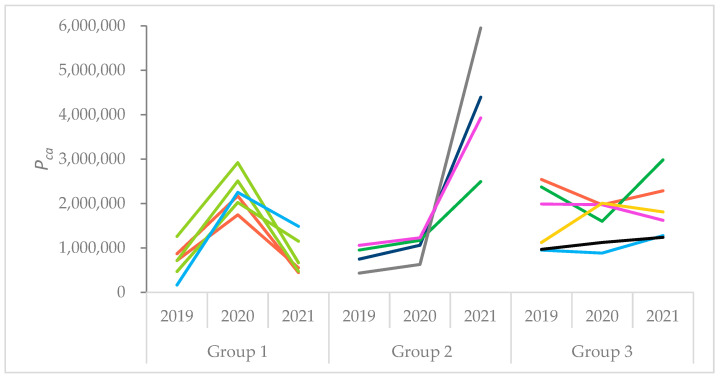
Pollen production per catkin (*P_ca_*) (*y*-axis) in 2019–2021 assessed for the selected non-topped trees at the seed planation Wildberg and categorized in three groups with similar temporal behavior. The color of the lines symbolizes trees of the same clone.

Due to the effects of topping, the assessment of synchrony in pollen production levels was carried out for all non-topped trees (*n* = 16) for which the temporal development was evaluated and classified into three different groups (Figure 5). The classification was performed visually according to the maximum in pollen production and the variation among years.

Group 1 includes the trees with maximum *P_ca_* in 2020 (*n* = 6). Group 2 consists of trees whose *P_ca_* was extraordinarily high in 2021 (*n* = 4). Group 3 has almost constant *P_ca_* values and/or minimum values in 2020 (*n* = 6). Only one clone (clone number 21) with three replications was always categorized to the same group (group 1). The trees of all other clones were distributed in more than one group.

These three different groups are highlighted in Figure 4 by different symbols. A one-way ANOVA did not reveal significant differences in cumulative solar radiation between the groups (F (2, 13) = 0.637, *p* = 0.545).

### 3.5. Differences within and among Clones

Based on the results that showed significant differences between topped and non-topped trees (Table 4), we selected five clones (12 trees) having at least two or more non-topped trees to further investigate the differences among clones, i.e., clone number 7 (*n* = 3), 21 (*n* = 3), 24 (*n* = 2), 30 (*n* = 2) and 42 (*n* = 2). The estimated pollen production of each year from those trees was analyzed to derive mean values and coefficient of variance (CV) (Table 5).

According to the calculated CV across all study years, clone 42 is the most consistent clone regarding *P_ca_* (CV = 0.57). Clone 42 was also found to produce the highest mean value for *P_ca_* and the lowest *C_su_* linked to the smallest CV (CV = 0.30). Similarly, clone 30 produced the lowest average pollen but was associated with a higher coefficient of variance (CV = 0.81) and a higher *C_su_* with a moderate coefficient of variation (CV = 0.60) compared to other clones.

Flowers per catkin (*fl*) were linked to lower CV values and was therefore most consistent compared to other reproductive metrics. Clone 7 had the lowest mean (96 *fl*) and CV (CV = 0.10) and clone 42 had the highest mean (127 *fl*). Clone 21 and clone 42 were linked to the highest CV (CV = 0.13) at the level of *fl*.

Interestingly, the CV is higher (for *fl*) and equal or higher (for *P_ca_*) when not splitted for each clone but when calculated for all the 16 non-topped trees (Table 5). Nevertheless, it is moderate in the case of *C_su_*.

**Figure 6:**
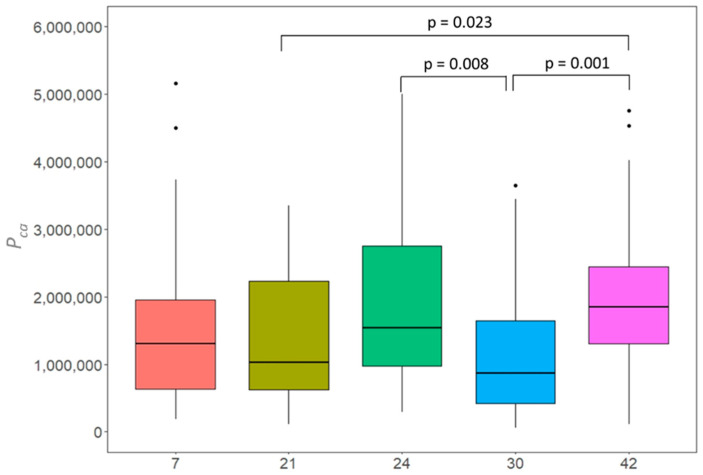
Boxplots based on pollen production per catkin (*P_ca_*) (eight replications per tree) estimated for five selected clones with at least two non-topped trees in the seed plantation in Wildberg for 2019, 2020 and 2021. Clones 7 and 21 consist of three trees each. Clone 24, 30 and 42 consist of two trees each. The interquartile range is represented by the height of the boxes, maximum and minimum values by the upper and lower whiskers, the median by bold horizontal lines in the boxes, points indicate outliers, lines above boxplots indicate pair of clones, which were significantly different (Kruskal–Wallis test and Dunn’s multiple comparison’s tests).

There was a statistically significant difference between *P_ca_* estimated for clones (H (4) = 21.44, *p* < 0.001) (see Figure 6). The post-hoc tests revealed that clone 30 was significantly different from clone 24 (*p* = 0.008) and clone 42 (*p* = 0.001). Clone 21 and clone 42 were also significantly different (*p* = 0.023).

For an average non-topped birch tree in the seed plantation, a mean crown volume was calculated as 81.55 m^3^ (average crown height = 6.28 m and average crown width = 4.98 m). This mean crown volume along with mean values of *C_su_* and *P_ca_* (in Table 5) were used to calculate mean number of catkins and mean *P_in_* for different clones and for an average birch tree (Table 6).

Table 6 demonstrates that clone 42 reproduces fewer catkins per tree (11,743) compared to other clones and 118 trees would be needed to produce the same amount of pollen produced by 100 average trees (based on mean values of all 16 trees). Clone 24 was found to produce almost the same number of catkins per tree as an average tree; however, it produces more pollen per tree. Therefore, 87 trees of clone 24 could produce the same amount of pollen as 100 average trees. Clone 30 produces a higher number of catkins per tree (25,443) and 92 trees would be needed to produce the same amount of pollen as 100 average trees.

## 4. Discussion

Our study investigating pollen production of 28 birch trees in three consecutive years is unique since we examined a large number of male birch inflorescences and assessed the internal variability of pollen production regarding genetic differences and similarities. In addition, this study excludes (major) environmental differences as well as age effects.

We estimated pollen production values at the level of catkins ranging from 48,000 pollen grains to 8.3 million pollen grains (mean 1.66 million). Some studies have already estimated pollen production values for *Betula pendula* (syn. *Betula alba*, *Betula verrucosa*). Erdtman [51] reported an estimate of 5.5 million pollen grains per inflorescence for *B. verrucose*. Jato et al. [30] estimated values ranging between 8.2 million and 4.8 million pollen grains per inflorescence, sampled from six trees of *B. alba* in northwestern Spain in 2002 and 2003, respectively. Piotrowska [43] estimated a mean value of 10 million pollen grains per inflorescence on the basis of 30 catkins deriving from three individuals. Although these studies have reported higher values compared to the mean *P_ca_* estimated in this study, they were based on either a few trees or estimated only for a single or two study years. Consequently, it is not known if sampling took place in a masting or non-masting years. For this reason, our study can be regarded as important since we have sampled 28 trees for three years and present a robust estimate for the mean pollen production of *Betula pendula*.

We found that birch catkins with fewer flowers produce more pollen and *vice versa*. This could be considered as an internal compensation since the plant aims at upregulating pollen production when the flower amount is low. Molina et al. [52] studied ten anemophilous species of aerobiological importance (*Betula* ssp. not included) and found a significant decrease in pollen per flower with a higher number of flowers per inflorescence. They suggested that there is a more or less constant amount (within a defined margin) for pollen production in anemophilous tree species. These species tend to compensate for reproductive characteristics (e.g., pollen per anther, flowers per tree, and inflorescences per tree) by increasing some and decreasing others. Our analysis showed that the number of flowers is the most homogenous value since a low coefficient of variance was associated to this measure, e.g., in clonal comparisons.

Our study shows an annual variation in pollen production with the lowest mean values in 2019 and the highest in 2021. Such alterations could be caused by yearly changes in the meteorological conditions of the locality. Some studies examining the relationship between temperature and pollen production suggested that warmer conditions result in higher pollen quantities. For example, experimental studies indicated that an increase in temperature [16] but also an increase in atmospheric CO_2_ concentration [16,53,54] was associated with a higher pollen production of common ragweed (*Ambrosia artemisiifolia*). However, it was also found that pollen production of birch (*Betula pendula* Roth) along an urban-rural gradient was negatively correlated with temperature [55]. The authors argue that the physiological performance of birch, which mainly grows at lower temperatures in mid to high latitudes, might be affected by (very) high temperatures and in turn react with a decrease in pollen production, as also suggested by Ziello et al. [56]. However, any differences in pollen production found in natural environments might also be affected by other factors, which attenuate or diminish the influence of temperature. In addition, the response to temperature might also be species-specific and strongly dependent on the methodologies used.

Although many studies have examined the temporal change in birch pollen concentrations based on pollen trap monitoring, there is no study presenting long-term changes in pollen production assessed using the same birch trees. Detecting the influence of temperature on pollen production based on the data presented in this study is not feasible, since (a) we only cover a period of three years and (b) a small spatial extent (1 ha) with similar temperature conditions, as documented using five installed temperature loggers. Many other environmental factors such as soil type and edaphic conditions as well as air pollutants are regarded to be similar as well. Especially the latter is also supposed to affect pollen production, as documented by Jochner et al. [55]. In their study, atmospheric NO_2_ levels were negatively associated to pollen production.

However, we found differences in solar radiation, which arise mainly from the forested surrounding of the seed plantation. During the study years, the correlations between pollen production and solar radiation did not vary much in magnitude, but they did shift in sign. Therefore, we calculated the correlation coefficient for mean (2019–2021) pollen production, but the association to solar radiation was no longer discernible. Thus, solar radiation, which is known to lead to higher stem and tissue temperatures [57] might also be inadequate to explain variations of pollen production at a small spatial scale. This was also evident when comparing solar radiation values with the association of birch trees to groups with similar pollen production patterns across the study years.

We did not detect a high synchrony of pollen production levels of birch trees within the birch plantation since we found that six trees exhibited the highest pollen production in 2020, four trees a very high pollen production in 2021 and six trees an almost constant pollen production across the study years. The birch trees allocated to one of these three groups did not necessarily belong to one clone. Thus, a coherence on the level of clones was not evident, except for one clone group.

Masting behavior, the inherent year-to-year variation in pollen production by plant populations [58,59], can be observed in several tree species [60,61] including birch [14,30]. Flowering and annual pollen sums in birch were reported to fluctuate from year to year [62]. Using aerobiological data gathered from pollen traps that assess the pollen concentration of the ambient outdoor air, a biennial [63] as well as triennial rhythm [64] of masting can be observed. Related to *Betula* species, Ranta et al. [59] found that male flowering shows synchronized annual fluctuations among stands at a regional scale; however, stand-specific catkin number during the masting year varies considerably, which in turn might also influence the pollen produced. This is also in accordance with our findings since the numbers of catkins varied (mean *C_s_* (SD) = 23 (8), 44 (26) and 19 (12) in 2019, 2020, and 2021, respectively, Table 2) within the plantation.

Asynchronous pollen production levels, which were found in our study might be caused by the resource balance of an individual tree. If the initial resource stock and the resource gained afterwards differ from one individual to the next in the stand, masting synchronization might not occur [65,66], even under the same environmental conditions [65]. In addition, plant-pathogen and plant-mycorrhizosphere interactions may reduce or enhance the impacts of abiotic stress on resource allocation [67] which could be specific to each tree.

Effects on pollen production and catkin formation were especially obvious two years after topping. Topping and pruning have been considered as adequate tree crown management techniques to enhance seed production, specifically in conifer seed orchards, or to promote the branching of the trees [47,68,69]. Viherä-Aarnio and Ryynänen [47] studied seed production of silver birch individuals that were topped in the second year in a greenhouse experiment. In the fourth year, a ten times higher amount of seeds per plant (compared to the previous year) was obtained. This was followed by a year with poor flowering and seed production. In our study, we cannot conclude on any effects in upcoming years; therefore, we recommend a longer monitoring of pollen production after topping in further studies.

Birch clones characterized by on average lower pollen production could be an opportunity to reduce the prevalence of allergies. In an experiment, transgenic birch grown in a greenhouse showed the ability to prevent flowering in silver birch trees [70]. However, such preventions might be associated with adverse side effects such as aberrant branching and growth disturbance. Therefore, we suggest selecting birch clones associated with low pollen production. We estimated *P_ca_* ranging between 1.17 million (clone 30) and 1.97 million (clone 42) pollen. Clones producing less pollen might contribute to lower pollen concentrations in the atmosphere. Therefore, clone 42 could be recommended for urban plantations. Similarly, clone 24 needs 87 trees to produce the same pollen amount as 100 average trees. This clone could be suitable in seed plantations to increase seed production. Since variations within clones were especially obvious when comparing pollen production levels across years (Chapter 3.4), we highly recommend monitoring pollen production for a longer term in order to create robust averages for different clones.

## 5. Conclusions

Our study revealed considerable differences in pollen, flower and catkin productions by birch trees among the study years. Moreover, we found topped birches were associated to higher reproductive outputs, especially two years after the intervention. We conclude that synchronicity of pollen production levels is not utterly site-specific and, in addition, not strictly dependent on genotypes. The detected variations in solar radiation within the plantation were found to be not responsible for asynchrony. Since we revealed significant differences in pollen production between clones, we propose that a wise selection of plants depending on their application (seed plantation, urban planting) might be advantageous. As these conclusions are based on three years of investigation, we recommend a longer monitoring period to further extend our knowledge related to pollen production of anemophilous tree species. In addition, further experimental studies with intended treatment such as pruning and topping under different climatic conditions are highly desirable.

## Figures and Tables

**Table 1:** Studied clones and their geographic origins.

Clone Number	Number of Individuals per Clone	Origin	Latitude (N)	Longitude (E)
55–04	1	Lahr	48°21′	7°52′
55–07	4			
55–10	4			
55–46	2			
55–21	4	Nürtingen	48°37′	9°20′
55–24	2	Karlsruhe	49°00′	8°24′
55–30	2	Kehl	48°35′	7°51′
55–33	1			
55–38	3	Kandern	47°43′	7°39′
55–42	3			
55–47	2	Villingen-Schwenningen	48°04′	8°24′

**Table 2:** Descriptive statistics of pollen production per catkin (*P_ca_*) and catkins per crown sampling unit (*C_su_*; 0.125 m^3^) (minimum, maximum, mean, median and standard deviation) estimated from 28 selected birch trees of the seed plantation Wildberg during 2019–2021.

Year	Minimum	Maximum	Mean	Median	Standard Deviation
Pollen production per catkin (*P_ca_*)					
2019	48,000	8,270,000	1,359,049	983,500	1,245,134
2020	108,000	4,172,000	1,511,170	1,360,000	892,862
2021	184,000	8,018,000	2,090,888	1,796,000	1,495,281
2019–2021	48,000	8,270,000	1,658,846	1,356,000	1,277,605
Catkins per crown sampling unit (*C_su_*)					
2019	10	45	23	22	8
2020	10	120	44	35	26
2021	3	60	19	20	12
2019–2021	3	120	29	23	20

**Table 3:** Spearman correlations between averaged reproduction metrics for all studied years and 28 birch individuals of the Wildberg seed plantation. *r_s_*: Spearman’s correlation coefficient, *p*: significance.

Reproductive Metrics	*P_fl_*		*P_ca_*		*fl*	
	*r*_s_	*p*	*r*_s_	*p*	*r*_s_	*p*
*P_ca_*	0.980	0.000				
*fl*	−0.230	0.000	−0.040	ns		
*C_su_*	−0.060	ns	−0.020	ns	0.200	0.000

**Table 4:** Reproductive metrics (mean and median) of topped (*n* = 12) and non-topped trees (*n* = 16) in 2020 and 2021 and comparisons (Mann–Whitney U test) between them.

Reproductive Metrics	Group	2020			2021		
		Mean	Median	*p*	Mean	Median	*p*
*P_ca_*	Topped	1,252,938	1,116,000	0.000	2,143,096	2,098,000	0.016
	Non-topped	1,704,844	1,654,000		2,048,469	1,486,000	
*P_fl_*	Topped	10,564	9,617	0.000	19,271	17,362	0.039
	Non-topped	15,935	14,742		19,266	13,309	
*P_cr_*	Topped	485,992,500	399,840,000	ns	405,397,846	306,200,000	0.000
	Non-topped	541,242,500	384,200,000		238,281,125	169,840,000	
*fl*	Topped	122	117	0.000	112	112	0.030
	Non-topped	112	112		107	107	
*C_su_*	Topped	53	45	0.009	23	20	0.002
	Non-topped	38	35		16	17	

**Table 5:** Mean values for reproductive metrics and associated coefficients of variance (CV) of clones with non-topped trees in the seed plantation Wildberg for 2019–2021.

Clone	*fl*	*P_fl_*	*P_ca_*	*C_su_*	*P_cr_*
7	96 (0.10)	15,366 (0.66)	1,478,347 (0.71)	27 (0.48)	333,661,556 (0.95)
21	106 (0.13)	13,164 (0.71)	1,354,889 (0.69)	29 (0.49)	385,906,000 (1.06)
24	98 (0.11)	20,362 (0.70)	1,929,146 (0.65)	25 (0.88)	331,433,333 (0.87)
30	120 (0.11)	9732 (0.81)	1,169,417 (0.81)	39 (0.60)	444,390,000 (1.26)
42	127 (0.13)	15,787 (0.54)	1,967,167 (0.57)	18 (0.30)	312,689,333 (0.79)
16 non-topped trees	112 (0.17)	14,994 (0.84)	1,612,250 (0.81)	26 (0.56)	332,198,375 (1.04)

**Table 6:** Mean number of catkins and mean pollen production for different clones and for an average tree. The last column shows the equivalence of the selected clones’ trees to an average tree.

Clone	Mean Catkins Per Tree	Mean Pollen Production per Tree (*P_in_*)	Equivalent to 100 Average Trees
Average tree	16,962	27,347,187,742	100
7	17,615	26,040,366,183	105
21	18,919	25,633,543,942	107
24	16,310	31,463,863,118	87
30	25,443	29,753,697,858	92
42	11,743	23,100,462,441	118

## Data Availability

Not applicable.

## Appendix A

**Table A1:** Descriptive statistics of pollen production per catkin (*P_ca_*), pollen production per flower (*P_fl_*), pollen production per volume unit of crown (*P_cr_*), flowers per catkin (*fl*) and catkins per crown sampling unit (*C_su_*_;_ 0.125 m^3^) (minimum, maximum, mean, median and standard deviation) estimated from 28 selected birch trees of the seed plantation Wildberg during 2019–2021.

Reproductive Metrics	Minimum	Maximum	Mean	Median	Standard Deviation
All years					
*P_ca_*	48,000	8,270,000	1,658,846	1,356,000	1,277,605
*P_fl_*	407	80,291	15,018	12,093	12,008
*P_cr_*	4,992,000	2,333,440,000	359,736,647	244,592,000	348,107,774
*Fl*	77	173	113	112	18
*C_su_*	3	120	29	23	20
2019					
*P_ca_*	48,000	8,270,000	1,359,049	983,500	1,245,134
*P_fl_*	407	80,291	12,001	8691	11,360
*P_cr_*	4,992,000	1,654,000,000	250,112,679	164,292,000	261,586,850
*Fl*	77	173	116	113	19
*C_su_*	10	45	23	22	8
2020					
*P_ca_*	108,000	4,172,000	1,511,170	1,360,000	892,862
*P_fl_*	788	37,250	13,633	12,093	8729
*P_cr_*	12,960,000	2,333,440,000	517,563,929	388,160,000	409,661,684
*Fl*	82	173	117	117	19
*C_su_*	10	120	44	35	26
2021					
*P_ca_*	184,000	8,018,000	2,090,888	1,796,000	1,495,281
*P_fl_*	2000	71,589	19,268	16,102	14,030
*P_cr_*	8,880,000	1,731,360,000	313,195,517	207,240,000	300,234,937
*Fl*	78	142	109	107	16
*C_su_*	3	60	19	20	12
